# Non-linear Relationships Between the Built Environment and Walking Frequency Among Older Adults in Zhongshan, China

**DOI:** 10.3389/fpubh.2021.686144

**Published:** 2021-08-05

**Authors:** Jiani Wu, Chunli Zhao, Chaoyang Li, Tao Wang, Lanjing Wang, Yi Zhang

**Affiliations:** ^1^State Key Laboratory of Ocean Engineering, China Institute for Urban Governance, Shanghai Jiao Tong University, Shanghai, China; ^2^Transport & Roads, Department of Technology and Society, Faculty of Engineering, Lund University, Lund, Sweden

**Keywords:** non-linear, built environment, walking frequency, older adults, thresholds

## Abstract

**Aim:** Promoting walking activity is an effective way to improve the health of older adults. Walking frequency is a critical component of walking behavior and an essential determinant of daily walking levels. To decipher the association between the built environment and walking frequency among older adults, this study's aims are as follows: (1) to empirically test whether non-linear relationships between the two exist, and (2) to identify the thresholds of the built environment characteristics that promote walking.

**Methods:** The walking frequency of old adults was derived from the Zhongshan Household Travel Survey (ZHTS) in 2012. The sample size of old adults aged 60 or over was 4784 from 274 urban and rural neighborhoods. A semi-parametric generalized additive model (GAMM) is used to analyze the non-linear or non-monotonic relationships between the built environment and the walking frequency among older adults.

**Results:** We found that non-linear relationships exist among five out of the six built environment characteristics. Within certain thresholds, the population density, sidewalk density, bus stop density, land use mixture, and the percentage of green space are positively related to older adults' walking trips. Furthermore, the land use mixture and the percentage of green space show an inverse “V”-shaped relationship.

**Conclusions:** Built environment features can either support or hinder the walking frequency among older adults. The findings in the current study contribute to effective land use and transport policies for promoting active travel among older adults.

## Introduction

Physical activity (PA) brings numerous positive health outcomes for older adults by lowering the incidence of chronic diseases, disability, and bone fractures (1). As the most common PA for older adults, walking is low-cost and low-risk, and easy to incorporate into daily life (2, 3). All else being equal, walking over 120 minutes per week is positively associated with a more negligible risk of all-cause mortality (4). Older adults are, in general, the least physically active group of the population (5). Therefore, encouraging walking among older adults is a crucial intervention to improve the quality of life and healthy aging (6).

According to the United Nations' latest report, the global population of older adults aged 65 years or over is estimated at 727 million in 2020. The number is projected to more than double by 2050, reaching over 1.5 billion (7). Between 2020 and 2050, Eastern and South-Eastern Asia will experience the most significant percentage increases in the older population. As the most populated country in Eastern and South-Eastern Asia, China's aging population is expected to increase from 180 million in 2020 to 380 million in 2050 (8). However, in a review on the built environmental correlates of older adults' walking (9), only 15% of the studies focused on Eastern and South-Eastern Asia countries with ultra-high residential density. Therefore, the findings in the Western context are not necessarily translatable to the Chinese context. Furthermore, few interventions supporting walking among older adults have been implemented in China, as little is known about the barriers and facilitators of their walking (10). Therefore, it is of great significance to study the walking behavior of old adults in the Chinese context.

The investigation into the effects of the built environment on older adults' walking behaviors has encouraged evidence-based, health-oriented interventions to promote walking activity among older adults (11). Older adults are more susceptible to the built environment because they are more inclined to travel short distances. Therefore, they need safe facilities and spend more time in parks (12). Previous studies have indicated that a walking-friendly built environment, compact and mixed urban form, connective pedestrian infrastructure, accessible commercial and service opportunities, and green spaces and parks are essential determinants of older adults' walking (6, 13–15). However, most literature assumes that the relationships between the built environment and walking are linear or at least linear in variables. The linkages between the built environment and walking behavior remain ambiguous and mixed (16). The impact of a built environment variable on walking behavior may differ when it falls into different ranges of the variable, and its effect may be saturated when it reaches a certain level (17–19). Scholars of land use, transport, and public health called for studies to investigate the non-linear relationships between the built environment and travel behavior (20). Using generalized additive mixed models, the non-linear effects of the built environment on older adults' walking have been implied by Cerin et al. (21). Ding et al. used a gradient boosting decision trees (GBDT) method and found strong non-linearity between the built environment and driving distance in an empirical study in Oslo, Norway (18). Ding et al. applied a gradient boosting logit model to substantiate a non-linearity relationship of ridership, with Metrorail data collected in the Washington Metropolitan Area (19). Using a gradient boosting decision trees (GBDT) algorithm, Tao et al. (22) explored the non-linear associations between spatial attributes and walking distance to public transport (22). Their studies acknowledge the complexity of the effects of the built environment on older adults' walking. The results suggest the potential benefits of exploring threshold effects to inform more effective environmental interventions. However, relevant exploration of the non-linear associations between the built environment and walking among older adults is somewhat limited.

With data collected from the Zhongshan metropolitan area in China, this paper employed a semi-parametric generalized additive mixed model (GAMM) to explore the potential non-linear relationships between the built environment and walking frequency among older adults. The present study contributes twofold to the existing literature. First, it facilitates understanding of the relationships between the built environment and active travel in a developing country with rapid urbanization and motorization. Second, it challenges the linearity assumption and identifies the non-linear associations between various built environment characteristics and walking frequency among older adults. The threshold effects of the built environment observed in the study provide nuanced insights for policymakers and planners on efficient land use and transport planning strategies and policies to encourage walking among older adults.

## Materials and Methods

**Figure 1:**
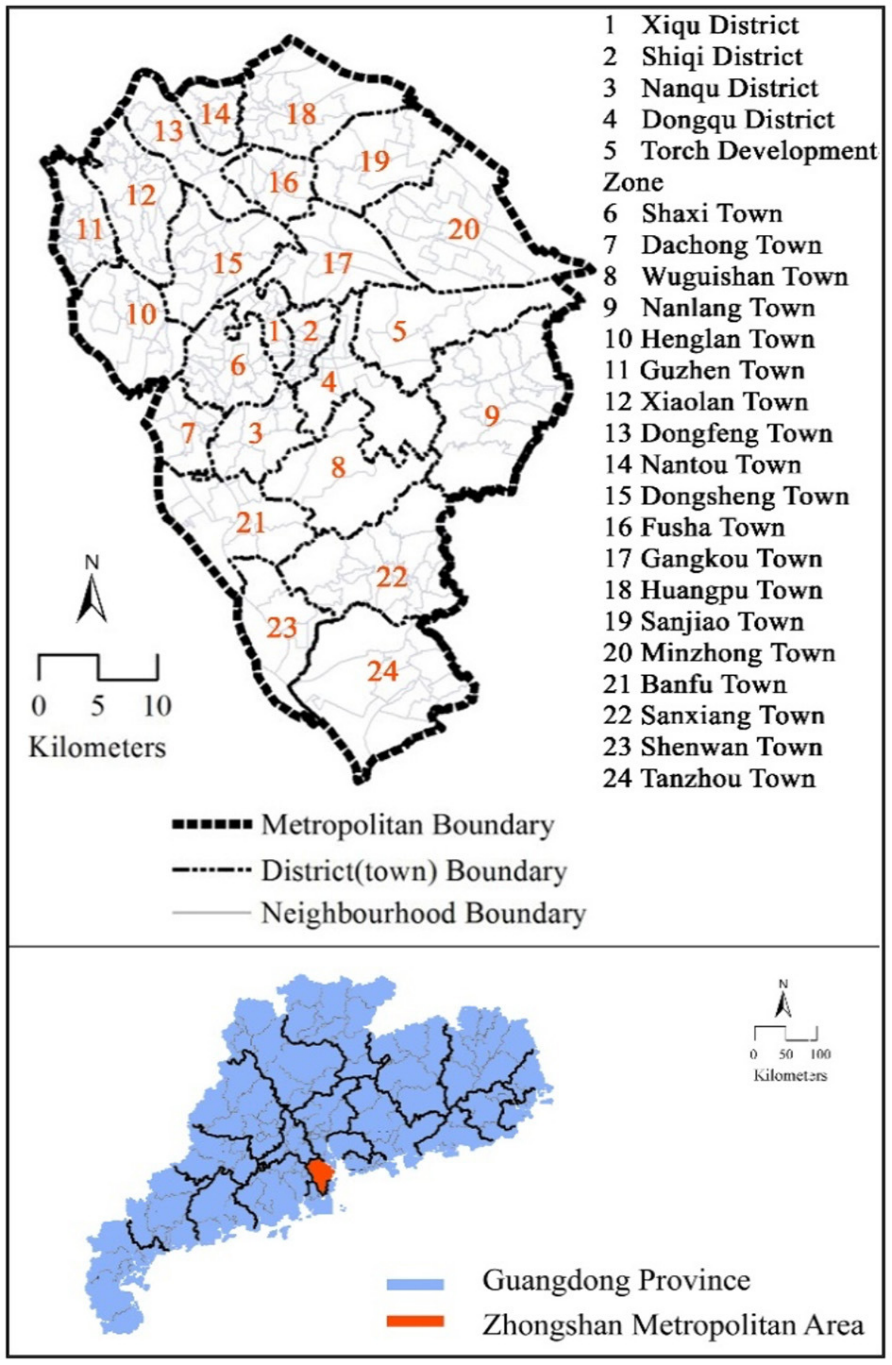
Study area. The upper side of the figure shows the city boundary, district (town) boundary, and the neighborhood boundary of Zhongshan; while the bottom half shows the location of Zhongshan in Guangdong Province.

This study chose Zhongshan Metropolitan Area, Guangdong Province, China, as a case to examine the walking frequency among older adults in the Chinese context. Zhongshan is a medium-sized prefecture-level city with 24 districts and towns (Figure 1). In the three largest coastal urban agglomerations with the most competitive economies in China, there are about 20 medium-sized cities with similar urbanization and motorization level and urban transport characteristics to Zhongshan (6). The walking frequency of older adults in Zhongshan averages 1.42 trips per day, covering 55% of all utilitarian trips. Utilitarian walking refers to walking trips undertaken to fulfill common purposes (e.g., accomplishing errands or getting to work) (23, 24).

### Data Collections

The walking activity data were obtained from the Zhongshan Household Travel Survey (ZHTS) in 2012. Selected by stratified random sampling, the sample size of older adults was 4,784 (2,905 male and 1,879 female) from 274 urban and rural neighborhoods, with a sample rate of 2%. The numbers of older adults aged 60–69, 70–79, and over 79 were 2,979, 1,078 and 272, respectively. The 2012 ZHTS provided a travel diary including self-reported walking activity, e.g., the frequency, duration, and purpose of walking trips, and the respondents' personal and household socio-demographics. The description of the population are shown in Table 1.

**Table 1:** Description of the population.

**Sample group**	**Range**	**Average**	**Median**	**STDEV**
Male and Female	60–95	67.05	65	6.61
Male	60–95	66.95	65	6.53
Female	60–95	67.22	65	6.87

### Variable Selection

Ewing and Cervero suggested that each D variable of the “five Ds” built environment contains several features commonly used in the built environment-travel behavior research (25). The “five Ds” are density, design, diversity, distance to transit, and destination accessibility. Additionally, the aesthetic is another built environment feature that influences walking frequency among older adults (14, 20). The meaning and represent variables (considering the best available data) of the five Ds and aesthetics are shown in Table 2.

**Table 2:** Dimensions of the built environment.

**Dimensions**	**Meaning**	**Represent variables**
Density	The variables of interest per unit of area	Population density, Residential density
Design	The characteristics of the street network inside an area	Sidewalk density, Road network density
Diversity	The number of different land uses in a fixed area and the represented degree	Land-use mixture
Distance to transit	The level of transit service at the residences or workplaces	Bus stop density
Destination accessibility	Ease of access to a certain location	Commercial accessibility, Distance to the city center
Aesthetic	Attractiveness and appeal of a place	The percentage of green space

This study carries out a Pearson correlation analysis to reduce the multicollinearity of independent variables and select the best variables. Multicollinearity refers to the high correlation between the independent variables in the linear regression model. It makes the model estimation distorted or difficult to estimate accurately. The Pearson correlation coefficient between two independent variables can be used to determine the degree of correlation. If the correlation coefficient between the variables is large, there is usually strong collinearity between them. Therefore, only one of the variables is selected as the independent variable for modeling. In Table 3, the Pearson correlation coefficient between residential density and sidewalk density is 0.843, showing a significant correlation. The correlations of the distance to the city center with other variables were higher than commercial accessibility. Therefore, the built environment variables finally selected in this study are population density, sidewalk density, land-use mixture, bus stop density, commercial accessibility, and the percentage of green space.

**Table 3:** Pearson correlation coefficient between explanatory variables.

	**Population density**	**Residential density**	**Sidewalk density**	**Bus stop density**	**Commercial accessibility**	**Distance to center**	**Mixture**	**Green-space**
**Population density**	1.000	1.000	0.500	0.488	−0.030	0.497	−0.352	0.112
**Residential density**	1.000	1.000	0.843	0.488	−0.030	0.600	−0.352	0.112
**Sidewalk density**	0.500	0.843	1.000	0.460	0.075	0.482	−0.132	0.126
**Road network density**	0.500	0.843	1.000	0.460	0.075	0.482	−0.132	0.126
**Bus stop density**	0.488	0.488	0.460	1.000	0.113	0.474	−0.008	0.100
**Commercial accessibility**	0.000	0.000	0.100	0.100	1.000	0.800	0.200	0.100
**Distance to center**	0.500	0.600	0.500	0.500	0.800	1.000	0.000	0.200
**Mixture**	−0.352	−0.352	−0.132	−0.008	0.238	−0.027	1.000	0.295
**Greenspace**	0.112	0.112	0.126	0.100	0.077	0.189	0.295	1.000

### Main Variables

The built environment data were collected based on neighborhoods, which are spatially equivalent to the traffic analysis zone (TAZ). As designed to be homogeneous concerning socio-demographic characteristics and living conditions, TAZs share boundaries with administrative divisions in most cases. According to the administrative divisions in Zhongshan, a total of 274 traffic analysis zones (neighborhoods) were selected in this study.

The population density, sidewalk density, bus stop density, and the percentage of green space are explained in Table 4. The land-use diversity represents the degree to which different land uses in a neighborhood are mixed. It is calculated by the Entropy Index (EI) (26), where 0 indicates single-use environments and 1 stands for the equalization of different land uses in area coverage. EI is defined by:

(1) $$\begin{array}{r} EI=\sum_{i=1}^{n}P_{i}\log\left(1/P_{i}\right)\ldots \ldots \end{array}$$

where *n* = number of unique land uses, *n* ≥ 1; *P*_*i*_ = percentage of land use *i*'s coverage over total land use coverage (27). Commercial accessibility describes the ease of access to commercial attractions. The neighborhood-level commercial accessibility is defined by the area of coverage of commercial facilities within a one-kilometer distance from the centroid of a neighborhood. The 2012 ZHTS shows that a travel distance of one kilometer covers 70% of older adults' home-based commercial trips. For each neighborhood, we first defined the centroid of each neighborhood as the origin and distributed a one-kilometer travel distance as a buffer to the main roads from the origin. Then we formed an enclosed area with the endpoints of the acceptable travel distances in ArcGIS. Finally, we collected the data of the area covered by commercial facilities in the enclosed area in ArcGIS. The dependent variable and independent variables (i.e., socio-demographics and built environment) used in this study are shown in Table 4.

**Table 4:** Description of variables used in the analysis.

**Variables**	**Description**	**Mean/percentage (%)**	**St.Dev**.
Dependent variables			
Frequency	Frequency of utilitarian walking trips of older adults, trips per day, count	1.42	1.43
Personal demographics			
Gender	Male	60.43	/
	Female	39.57	
Age	Age of the respondent in years, count	67.05	6.61
Employ	The respondent is employed	24.46	/
	The respondent is non-employed	75.54	
Prowalk	The respondent who favors walking over other modes	26.82	/
	The respondent who favors other modes over walking	73.18	
Household demographics			
HH-1	Household size is one person	26.82	/
HH-2	Household size is two persons	35.34	/
HH>2	Household size is over two persons	37.84	/
Highinc	Household income is high (>60,000 RMB/yr)	15.25	/
Midinc	Household income is medium (20,000–6,000 RMB/yr)	47.82	/
Lowinc	Household income is low (<20,000 RMB/yr)	36.93	/
Bikes	Number of bikes in a household, count	0.61	0.71
E-bikes	Number of electric bikes in a household, count	0.22	0.46
Motors	Number of motorcycles in a household, count	0.76	0.85
Cars	Number of private cars in a household, count	0.17	0.44
Built environment variables at the traffic analysis zone (TAZ) level			
(Explanatory variables)			
Popden	Population density,1000 persons/km^2^, continuous	8.08	10.23
Mixture	Land use mixture, Entropy Index, continuous	0.7	0.18
Sidewalk	Sidewalk density, km/ km^2^, continuous	4.65	3.31
Busstop	Bus stop density, number of bus stops per km^2^, continuous	1.78	2.50
Comacc	Area coverage of commercial establishments within 1 km from the center of a neighborhood, in ha, continuous	33.19	33.08
Green	Percentage of green space land use among all land uses, continuous	0.07	0.08

*Sample size = 4329*.

### Modeling Approach

The relationship between the built environment and walking trips of older adults is complex, uncertain, and probably non-linear (28). The semi-parametric GAMM as penalized generalized linear models can relax the pre-defined relationship (e.g., linear or log-linear) between the utility and explanatory variables (29). This study adopted a semi-parametric GAMM to disentangle the non-linear or non-monotonic relationships (17, 26). The model relating a univariate response variable μ to a set of explanatory variables x_1_, x_2_, …, has the general form:

(2) $$\begin{array}{r} g(\mu )=s_{0}+\sum {}_{i=1}^{r}\;\beta_{i}X_{i}+\sum {}_{j=1}^{p}\;s_{j}(X_{j})+\varepsilon \end{array}$$

where *g* is a smooth monotonic “link function” that allows a valuable degree of non-linearity into the model structure (27). *s*_0_ is the intercept, μ is the dependent variable, which is the daily utilitarian walking trips (times/day) among older adults. *X* is the independent variable, *X*_*i*_ represents the household and personal socio-demographic variables (i.e., gender, age, household size, employment status, household income, ownership of bikes, e-bikes, motorcycles, cars), *X*_*j*_ stands for the built environment variables (i.e., population density, land use mix, sidewalk density, bus stop density, commercial, and percentage of green space). *s*_*j*_(*X*_*j*_) are smooth functions of built environment variables. With the smooth term, it becomes a semi-parametric model with a parametric component and a non-parametric component. The parametric part is $\overset{r}{\underset{i=1}{\sum }}\beta_{i}X_{i}$, and the non-parametric part is $\sum_{j=1}^{p}s_{j}(X_{j})$. ε is the residual of the model and ε ~ *N*(0, σ^2^). The details of the link function and smooth function estimation process were presented in Appendix A.

## Results

Employing the semi-parametric GAMM described in the section of the modeling approach, we developed a model to investigate significant correlates of utilitarian walking frequency among older adults and illustrate the non-linear effects of relationships with the built environment. Model estimation results are presented in Table 5.

**Table 5:** Results of the semi-parametric generalized additive mixed model.

**Parametric variables**	**Coefficients**	**T-statistic**	***P*-value**
Intercept	−0.18	−1.27	0.20
Personal demographics			
Gender	−0.13	−4.98	*p* < 0.01
Age	−0.01	−4.95	*p* < 0.01
Employ	−0.20	−4.89	*p* < 0.01
Prowalk	0.47	17.42	*p* < 0.01
Household demographics			
Hh.1	−0.10	−2.12	0.03
Hh.2	−0.07	−1.75	0.08
Highinc	0.08	1.75	0.08
Midinc	−0.03	−0.91	0.36
Bikes	−0.11	−4.98	*p* < 0.01
Ebikes	−0.20	−4.98	*p* < 0.01
Motors	−0.07	−3.13	*p* < 0.01
Cars	−0.06	−1.81	0.07
**Non-parametric variables**	**Estimated degree of freedom**	**F-statistic**	***p*** **-value**
Built environment variables			
Popden	3.15	3.42	*p* < 0.01
Mixture	2.25	4.12	0.02
Sidewalk	2.88	7.99	*p* < 0.01
Busstop	2.17	6.95	*p* < 0.01
Comacc	1.00	3.18	0.08
Green	2.83	13.20	*p* < 0.01
Model fit measurements			
Adjusted R^2^	0.21		
Deviance explained (%)	21.2%		
Generalized Cross-Validation	1.60		
Scale est.	1.59		

*After removing the invalid data, the final sample size in the study is 4329*.

### The Associations Between Demographics and Utilitarian Walking Trips Among Older Adults

Table 5 presents the associations between personal and household socio-demographics and walking frequency among older adults. It is worth noting that the coefficients of parametric variables in a semi-parametric GAMM represent those in a traditional logit model. In general, socio-demographic variables are significantly correlated to walking frequency among older adults. The results show that the female walks more than the male, which is consistent with Kwan's “family responsibility hypothesis.” In addition, women often participate in more daily family activities, generating more daily trips, including walking. Moreover, the employed older adults walk less than the pensioners. Presumably, walking is not a desirable option for commuting trips (6, 30). Unsurprisingly, being younger or having a positive attitude toward walking is associated with more walking trips (31, 32).

Owning private vehicle instruments, especially bikes, e-bikes, or motorcycles, shows a negative association with walking frequency. Walking frequency among older adults is especially negatively correlated to bikes, e-bikes, and motorcycles. It demonstrates that walking is an alternative to those modes in certain travel distances. Furthermore, e-bike ownership has more significant marginal effects than bike ownership and motorcycle ownership. That implies that e-bike is a stronger competitor for walking than conventional bikes and motorcycles (6, 17). One possible reason is that e-bike is easy to operate, safe, comfortable, and free of exhaust gas and noise pollution. It substitutes for conventional bikes, motorcycles, transit, and cars and meets the needs of the working class and middle-older-aged adult (17, 33).

### Non-linear Relationships With Built Environment Variables

**Figure 2:**
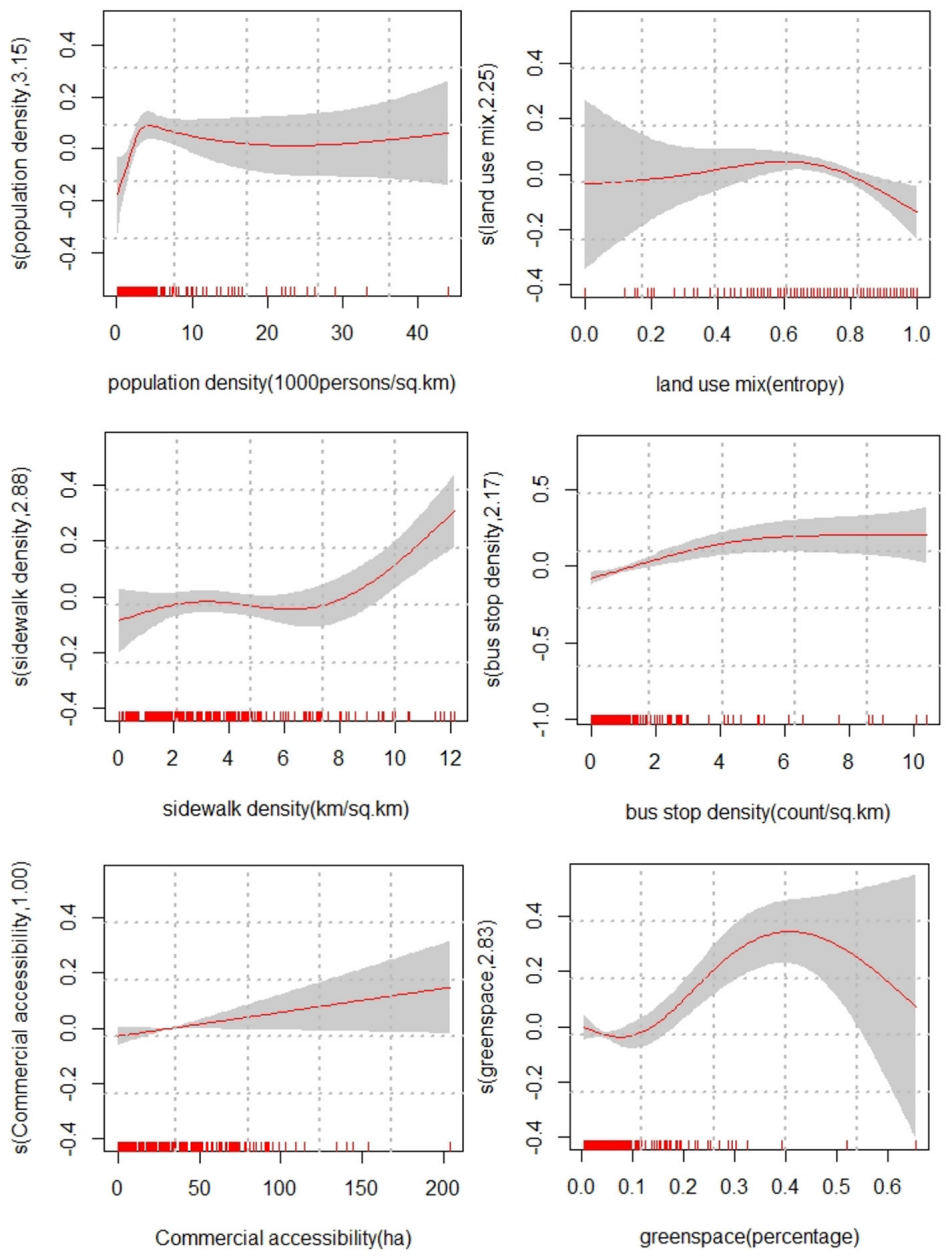
The relationships between built environment variables and walking frequency among old adults. The numbers shown on the vertical axis in each plot indicate the estimated degrees of freedom. Solid lines represent the estimated effects of built environment variables. Shaded regions denote the 95% confidence intervals. The estimated curves are centered around zero because of the identifiability constraints.

This study treated built environment features as non-parametric variables to disentangle their non-linear effects. In Table 6, the estimated degree of freedom (EDF) of commercial accessibility is 1.00, suggesting a stronger linear association, consistent with the plot in Figure 2. However, the EDFs of the other five built environment variables range from 1.38 to 3.15, indicating non-linearity.

**Table 6:** Comparison between semi-parametric GAMM and linear regression.

**Variable**	**Semi-parametric GAMM**	**Linear regression**
	**Coefficients**	**Coefficients**
**Personal demographics**		
Gender	−0.13	0.04
Age	−0.01	0.00
Employ	−0.20	−0.01
Prowalk	0.47	0.28
**Household demographics**		
HH-1	−0.10	0.00
HH-2	−0.07	−0.00
Highinc	0.08	0.08
Midinc	−0.03	0.03
Bikes	−0.11	−0.05
E-Bikes	−0.20	−0.04
Motors	−0.07	0.01
Cars	−0.06	0.23
**Built environment variables**		
Popden	3.15	−0.01
Mixture	2.25	−0.36
Sidewalk	2.88	0.03
Busstop	2.17	0.01
Comacc	1.00	0.00
Green	2.83	0.31

When the population density is smaller than 7,000 persons/km^2^, it was positively correlated with the walking frequency among older adults. This positive association is somewhat congruent with the literature (34, 35). As population density increases, walking may become a desirable option among older adults. Higher population density tends to support walking-friendly street space and infrastructure, and various services and shops, which has an indirect influence on walking (35). However, beyond 7,000 persons/km^2^, the population density has a trivial marginal effect on walking frequency.

When the land use mix is smaller than 0.65, it was positively associated with the walking frequency among older adults, consistent with previous literature (36, 37). This relationship suggests that abundant commercial, service, and employment opportunities within walking distance promote older adults to walk more. By contrast, the land-use mixture seemed to affect the walking frequency negatively after the threshold of 0.65.

When the sidewalk density is below 6 km/km^2^, it had a limited marginal effect on the walking frequency. However, above the threshold of 6 km/km^2^, the sidewalk density showed a positive effect. This result makes sense because a denser sidewalk network indicates more walking-route choices, a walking-friendly environment, and better street connectivity, which increases older adults' willingness to walk (14, 38–40). These recommended values are significant to improving the cost-effectiveness of land use interventions.

When the bus stop density is within the range of six stops/km^2^, it showed a positive association. Walking is a desirable and prevalent travel option to connect trip origins and destinations with bus stops. More accessible bus stops are potentially correlated to increasing transit use as well as walking trips. However, when the density exceeds six stops/km^2^, the effect is saturated. The possible reason is that it may be hard to increase transit use by extra bus stops in neighborhoods with already high-level bus service provision. Hence, the increase in walking trips is minuscule.

The percentage of green space land use depicts an inverse U relationship with the walking frequency among older adults. In the range of 8–40%, the green space showed a positive association, consistent with the literature on green space and physical activity (6, 41, 42). Therefore, providing abundant green space in a neighborhood could be a desirable option to promote walking among older adults.

### Model Comparison With Linear Regression

Zhang et al.'s study (6) is among the earliest attempts on the built environment and the walking behavior among older adults in the Chinese context. Zhang et al. (6) adopted a zero-inflated Poisson regression and assumed that all built environment variables have exponential correlations with walking frequency and duration among older adults. This study employed the same dataset and independent variables as Zhang et al.'s previous work. Therefore, we compared the coefficients of the variables between semi-parametric GAMM and linear regression (Table 6). The assumption of linearity in variables in the zero-inflated Poisson regression renders the non-linear relationship unobservable.

## Discussion

### Major Findings

Using the 2012 Zhongshan Household Travel Survey (ZHTS) data, we examined the relationships between the built environment and walking frequency among older adults in Zhongshan, China. The walking frequency analyzed in the study is utilitarian walking trips per day. The older adults focus on adults aged 60 and over, in line with the definition of the aging population from the Law of the People's Republic of China on Protection of the Rights and Interests of Older Adults. Personal and household attributes related to gender, age, employment status, and attitudes toward walking are significantly connected to older adults' walking frequency. Specifically, being female or aged 60–69, or retired, or favoring walking over other modes is associated with more walking trips. The findings are consistent with previous literature (30, 31).

This study has shed light on the non-linear associations between the built environment and walking frequency among older adults. The non-linear patterns are demonstrated to be prevalent and vary among built environment attributes. The non-linear relationship shows that the impact of a built environment variable on walking behavior may differ when it falls into different ranges of the variable. Its effect may be saturated when it reaches a certain level (11, 17, 43). Specifically, in Zhongshan, the population density of 7000 persons/km^2^ and the land use mixture index of 0.65 is sufficient to promote walking among old adults. However, the ultra-densely populated areas and excessively mixed land use may negatively impact the frequency of older adults walking. This finding is in line with Cerin et al.'s work (21) that additional residential density in highly compact areas would not increase but even decrease the odds of walking among older adults. Thus, the association between population density and walking frequency is non-linear and different from Zhang et al.'s study in 2014 (6). This difference indicates the limitations of the linear assumption widely used in previous studies.

For land-use mixture, recent studies indicate that in dense Eastern-Asian cities (e.g., Seoul, Hong Kong), when the mixture is beyond a certain level, a decrease in walking frequency is observed (21, 44). Presumably, due to higher proximity to services and destinations, people tend to have trip-chains of multiple trips in a single journey (38, 45). However, the in-depth reasons behind the negative association require further study. Strategies for designing street networks in Zhongshan or similar cities should also account for the non-linear relationships found in this research. Above the threshold of 6 km/km^2^, sidewalk density shows a positive relationship with walking frequency. This finding is in line with the recommendations in China's Design Guidelines for the Pedestrian and Cycling Transportation System (46). The guideline recommended 6–10 km/km^2^ of sidewalk density as the baseline for creating walkability in neighborhoods. Moreover, for neighborhoods where walking is highly intensive, the desirable sidewalk density is 10 to 20 km/km^2^. Access to public transport fosters walking. Therefore, the density of bus stops has a significant impact on achieving regular engagement in walking among older adults. The bus stop density of six stops/km^2^ is sufficient to optimize walking frequency, consistent with previous studies (6, 22).

Greenery, street trees, parks, and green corridors provide a more pleasant and safer walking environment, contributing to the increased walking trips among older adults (47, 48). In Zhongshan, the effective range of green space land use for increasing walking among older adults is between 8% and 40%. When the percentage of green space land use exceeds 40% (two out of 274 neighborhoods fall in the category), the walking frequency decreases, consistent with the literature of Christiansen L B et al. (48). The reason is that the two neighborhoods are located in the city parks and nearly 20 km away from downtown. Therefore, the commercial and service establishments in the two neighborhoods are scarce and beyond the suitable walking distance for older adults.

It is worth mentioning that the present study employed the same dataset and independent variable as in Zhang et al.'s work (6). However, the two studies are significantly different in modeling approach and contribution. Zhang et al. (6) adopted a zero-inflated Poisson regression and assumed that all built environment variables have exponential correlations with walking frequency and duration among older adults. That research design is widely used in the existing literature. However, the present study employed a semi-parametric GAMM, hypothesizing that built environment variables have non-linear associations with the walking frequency among older adults. The results supported the hypotheses and challenged the conventional assumption used in Zhang et al.'s work (Table 6). Furthermore, it confirms that linear regression cannot reveal the complex non-linear associations between built environment variables and the walking frequency among older adults, as shown in Figure 2.

### Limitations

The study has a few limitations. First, the cross-sectional data reveals more associations than casual effects. Future studies are suggested to employ panel data or attitudinal attributes to disentangle the causal influences of the built environment on walking behavior. Second, this study does not subdivide age groups, genders, and urban/rural areas. It would be useful to examine how different ages, genders, and urban/rural areas affect the walking behavior of older adults. Third, the thresholds found in Zhongshan may not be transferable for other regions with different sizes or built environment features. The GAMM model used in this study with such smooth functions relaxes the assumption of linear or known parametric functional forms for the relationships which can potentially capture (even complex) non-linear functional forms. However, the modification of just one of the assumptions might have easily led to different estimations. Therefore, there are several uncertainties when using in different cities. We encourage planners to identify specific thresholds in land use planning for older adults according to studies in different regions.

## Conclusions

This study explores the non-linear relationships between the built environment and walking frequency among older adults with a semi-parametric generalized additive mixed model. The contributions of the study are threefold.

First, the findings suggest that the non-linear relationships between the built environment and walking frequency among older adults are prevalent. Based on the model results, the five built environment characteristics showing non-linearity are population density, land use mixture, sidewalk density, bus stop density, and the percentage of green space land use. The only linear built environment variable is commercial accessibility. The results are in line with recent literature (17, 22) and challenge the conventional linearity assumption in active travel studies. We also compared the present study with Zhang et al.'s work in 2014 using the same dataset but a zero-inflated Poisson regression (6). The assumption of linearity in variables in Zhang et al.'s work renders the non-linear relationship unobservable. Therefore, the linearity assumption of the regression model is flawed.

Second, the results underscore the critical role of the built environment in older adults' walking activity. Among the six built environment variables tested here, five were significantly associated with the walking frequency of older adults. In specific ranges, all else being equal, dense and mixed land use development, adequate pedestrian infrastructure, convenient bus service, and ample green land space are effective in encouraging older adults to opt for walking. Although the findings may not be directly used in other regions, the model developed in this study could be applied with their local data and facilitate strategies for land use and transport planning.

Third, the results suggest that built environment attributes have apparent threshold effects on the walking frequency among older adults. A built environment attribute may not have equivalent effects across the whole range of that attribute and thus finding the appropriate range would be cost-effective. In Zhongshan, the population density of 7,000 persons/km^2^ is sufficient to optimize walking frequency among older adults. Therefore, it is important to develop neighborhoods with relatively balanced age structures and avoid overly aggregating the elderly population. Moreover, the sidewalk density of 6 km/km^2^ should be the baseline in street network planning for creating walkability in neighborhoods. To promote walking activity among old adults, a bus stop density of 6 stops/km^2^ and a land-use entropy index of 0.65 are suitable as built environment interventions. Furthermore, for the percentage of green space land use, the effective ranges for increasing walking among older adults are between 8% and 40%. These findings can inform planners of the most effective ranges for planning.

## Data Availability Statement

The dataset presented in this article are not readily available because it belongs to the Zhongshan Municipality Natural Resources and Planning Bureau and is a part of the ongoing projects (Grant No. 18BSH143 of the National Social Science Foundation of China, Grant No. 20692109900 of Shanghai Science and Technology Program, and Grant No. 2020-APTS-04 of APTSLAB). Therefore, the dataset is confidential during this period.

## Ethics Statement

Ethical review and approval were not required for the study on human participants in accordance with the local legislation and institutional requirements. The participants provided their written informed consent to participate in this study.

## Author Contributions

JW conceived of the study and participated in its design and coordination. CZ and YZ led the manuscript preparation. LW and CL contributed to data collection and analysis. TW and YZ contributed to data collection. All authors read, contributed to, and approved the final manuscript.

## Conflict of Interest

The authors declare that the research was conducted in the absence of any commercial or financial relationships that could be construed as a potential conflict of interest.

## Publisher's Note

All claims expressed in this article are solely those of the authors and do not necessarily represent those of their affiliated organizations, or those of the publisher, the editors and the reviewers. Any product that may be evaluated in this article, or claim that may be made by its manufacturer, is not guaranteed or endorsed by the publisher.

## Appendix A

**The link function and smooth function estimation process**

This section describes the estimation approach of the link function and smooth function of the semi-parametric model. The semi-parametric GAMM uses a link function to establish a relationship between the dependent variable and a smoothed function of the independent variables. The strength of these models is their ability to deal with highly non-linear and non-monotonic relationships between the dependent and the set of independent variables. The link function is defined by the distribution of the response variable (29). The relationship between them is shown in Table A1.

**Table A1:** Distribution of response variable and link functions.

**Dependent variable distribution family**	**Link function**
Normal distribution	Identity link: g(Z) = Z
Binomial distribution	Logit link: g(Z) = log Z/(1-Z)
Negative binomial distribution	Inverse link: g(Z) = 1/Z
Gamma distributions	Log link: g(Z) = log(Z)
Poisson distributions	Log link: g(Z) = log(Z)

Misleading conclusions may be reached if an incorrect choice of the link is made (49). To avoid or, at least, mitigate the risks of such a wrong choice, predicting the dependent variable distribution is required. In this study, the dependent variable is walking trips, which is the continuous variable. Statistical literature has proposed that it is best to use the Kolmogorov-Smirnov (K-S) test to analyze whether a variable conforms to a certain distribution or compare whether there are significant differences between the two groups (50–52). Therefore, this study uses the K-S test to compare the actual frequency distribution with Gaussian, Uniform, Poisson, Exponential, etc. When assuming the response variable obeys gamma distribution, the results of the K-S test are D = 0.824, *P*-value = 0.102 > 0.05. The assumptions are established (53, 54). Finally, we selected the Log link for the link function.

In the semi-parametric GAMM model, it is necessary to consider both the goodness of fit and the smoothness of the curve. After experimental tests and research, we found that the spline-based smooth functions can automatically determine the optimal shape of the non-linearity. Therefore, this study uses the spline-based smoothing function, which has the following general form (27):

(A1) $$\begin{array}{l} f\left(x\right)=\;\overset{R}{\underset{r=1}{\sum }}\lambda_{r}S_{r}\left(x\right) \end{array}$$

where S(x) is a basic function, r is the number of basic functions, λ is a smoothing parameter to be estimated (27).

The spline is a continuous function piecewise-defined by polynomials and is widely used to smooth observed data (28). Various functions (e.g., thin plate regression splines, cubic regression splines, and P-splines) can be used as the specified spline basis function. The smoothing parameters are chosen by Generalized Cross-Validation (GCV). The smaller the GCV value, the better fit the model (27). This study chose thin plate regression splines because the GCV value is smallest, ‘knot locations' do not have to be selected and computation time is relatively efficient (17, 27).

In this study, the model estimation was implemented using the “mgcv” package in R (27).
